# Type 1 Diabetes and Autoimmune Thyroid Disease—The Genetic Link

**DOI:** 10.3389/fendo.2021.618213

**Published:** 2021-03-10

**Authors:** Lara Frommer, George J. Kahaly

**Affiliations:** Molecular Thyroid Research Laboratory, Department of Medicine I, Johannes Gutenberg University (JGU) Medical Center, Mainz, Germany

**Keywords:** type 1 diabetes, autoimmune thyroid disease, genetic link, susceptibility genes, HLA antigens, single nucleotide polymorphisms, autoimmune polyendocrinopathy

## Abstract

Type 1 diabetes (T1D) and autoimmune thyroid disease (AITD) are the most frequent chronic autoimmune diseases worldwide. Several autoimmune endocrine and non-endocrine disorders tend to occur together. T1D and AITD often cluster in individuals and families, seen in the formation of autoimmune polyendocrinopathy (AP). The close relationship between these two diseases is largely explained by sharing a common genetic background. The HLA antigens DQ2 (DQA1*0501-DQB1*0201) and DQ8 (DQA1*0301-DQB1*0302), tightly linked with DR3 and DR4, are the major common genetic predisposition. Moreover, functional single nucleotide polymorphisms (or rare variants) of various genes, such as the *cytotoxic T-lymphocyte- associated antigen (CTLA4)*, the *protein tyrosine phosphatase non-receptor type 22 (PTPN22)*, the *interleukin-2 Receptor (IL2Ra)*, the *Vitamin D receptor (VDR)*, and the *tumor-necrosis-factor-α (TNF)* that are involved in immune regulation have been identified to confer susceptibility to both T1D and AITD. Other genes including cluster of differentiation *40 (CD40)*, the *forkhead box P3 (FOXP3)*, the *MHC* Class I *Polypeptide-Related Sequence A (MICA)*, *insulin variable number of tandem repeats (INS-VNTR)*, the *C-Type Lectin Domain Containing 16A (CLEC16A)*, the *Erb-B2 Receptor Tyrosine Kinase 3 (ERBB3)* gene, the *interferon-induced helicase C domain-containing protein 1 (IFIH1)*, and various cytokine genes are also under suspicion to increase susceptibility to T1D and AITD. Further, *BTB domain and CNC homolog 2* (*BACH2)*, *C-C motif chemokine receptor 5* (*CCR5)*, *SH2B adaptor protein 3* (*SH2B3)*, and *Rac family small GTPase 2* (*RAC2)* are found to be associated with T1D and AITD by various independent genome wide association studies and overlap in our list, indicating a strong common genetic link for T1D and AITD. As several susceptibility genes and environmental factors contribute to the disease aetiology of both T1D and AITD and/or AP subtype III variant (T1D+AITD) simultaneously, all patients with T1D should be screened for AITD, and vice versa.

## Epidemiology and Serology

Type 1 diabetes (T1D) and autoimmune thyroid disease (AITD) are two frequent autoimmune endocrine disorders. The prevalence of T1D is increasing worldwide and has nearly doubled in the past 40 years, with a prevalence of 9.5 per 10,000 people worldwide. The incidence worldwide is 15 per 100,000, ranging from 0.1 per 100,000 in China and Venezuela to 40.9 per 100,000 in Finland and in Arab countries between 2.4 in Oman and 29.0 in Saudia Arabia (1, 2). The highest incidence rates are at ages 5 to 9 years in girls and 10 to 14 in boys. The gender ratio is almost balanced with a slight preponderance of males (3–7). AITD, encompassing Graves’ disease (GD) and Hashimoto’s thyroiditis (HT), is defined as the presence of positive thyroid-related Ab. It is most commonly associated with other autoimmune glandular and non-glandular disorders. AITD peaks in the fourth decade for GD or fifth and sixth decade for HT. AITD is frequently combined with T1D in populations of various ancestries, occurring together more often than expected by the population prevalence of each disease GD is less prevalent than HT, only affecting approximately 1–1.5% of the general population, but is the underlying cause of 50–80% of cases of autoimmune hyperthyroidism. While subclinical hyperthyroidism can be diagnosed in only 0.12% of the non-diabetic population, it is prevalent in 6–10% of T1D patients (8–10).

The association of at least two autoimmune-induced glandular disorders is defined as autoimmune polyendocrinopathy (AP). Several non-endocrine autoimmune diseases can also be present (11, 12). AP is divided into a very rare, monogenic juvenile type I and one polygenic adult type II with three variants or subtypes, which are distinguished according to age of presentation, disease combinations, and modes of inheritance (13–17). The most prevalent AP type is the disease association of T1D and AITD in the same individual defined as subtype III variant. While the AP juvenile type has an annual incidence of 1–2:100,000, prevalence numbers are 1:600–1:900, 1:4,000, and 1:25,000 in Iranian Jews, Italians, and Finns, respectively. The adult AP form is far more common, with a worldwide incidence and prevalence of 1.4–4.5:100,000 and 14–45:1,000,000, respectively. Due to the high number of cases remaining unreported, the prevalence may approximate 1:20,000 (11, 18, 19). No accumulation in individual ethnic groups that deviate from the worldwide prevalence is known. The manifestation peak of adult AP is in the fourth-to-fifth decade depending on the combination of endocrine components with a female predominance of 75% (16, 20). T1D is the main cost driver in AP (21). The inheritance pattern seems to be autosomal dominant with incomplete penetrance, while several genetic loci may interact with environmental factors, such as deficiency of vitamin D and selenium, high iodine intake, and exposure to irradiation. The exact underlying pathogenic mechanisms are however not yet completely characterized. Also, chemical contaminants such as polybrominated diethyl ethers, polychlorinated biphenyls and their metabolites, binding to thyroid transport proteins and disrupting the thyroid function by displacing thyroxine, may be involved (22).

T1D is a T-cell mediated chronic disorder characterized by the loss of insulin-producing pancreatic β-cells and the appearance of insulitis. Biomarkers for T1D are autoantibodies (Ab) to islet cell antigens (ICA), tyrosine phosphatase (IA2), glutamic acid decarboxylase-65 (GAD), insulin (IAA), and zinc transporter ZnT8Solute carrier family 30 member 8 (SLC30A8). GD is defined by the presence of autoimmune-induced hyperthyroidism together with the presence of thyrotropin receptor autoantibodies (TSH-R-Ab) in general and stimulatory TSH-R-Ab (TSAb) in particular (23–29). HT is defined as primary hypothyroidism with an atrophic thyroid gland, an increased serum level of serum thyroid peroxidase (TPO) Ab and/or blocking TSH-R-Ab (TBAb) (24, 26, 30–32). As many as 25% of adolescents with T1D have thyroid-related Ab (15, 33–36). Long-term follow-up suggests that 30% of patients with T1D will develop AITD (10, 11, 15). TPO-Ab are present in 15–30% of adults and in 5–22% of children with T1D, while only 2–10 and 1–4%, respectively, are present in healthy controls (10, 19, 37). Up to 50% of TPO-Ab positive T1D patients develop an AITD. The diagnosis of AP III variant involves serological measurement of organ-specific Ab and subsequent functional testing of baseline thyrotropin (TSH), follicle-stimulating hormone (FSH), luteinizing hormone (LH), free triiodothyronine (fT_3_) and thyroxine (fT_4_), estradiol, cortisol, testosterone and fasting morning glucose as well as serum sodium (Na^+^), potassium (K^+^), and calcium (38).

Numerous reports on the individual susceptibility genes for T1D and/or AITD have been published, however scarce data are available pertaining to joint susceptibility. Therefore, in the present review we focus on the genetic link between these two clinically relevant endocrine autoimmune diseases and outline the underlying pathogenetic mechanisms causing AP.

## Pathogenesis

In glandular autoimmunity, auto-aggression is considered multifactorial (39, 40). Antigen presenting cells (APC), such as ubiquitous dendritic cells, initiate the principal antigen-specific immune response (41). In non-lymphoid organs, immature dendritic cells pick up and fracture antigen molecules to subsequently migrate to the secondary lymphoid organs presenting their HLA class I or II associated antigen fragments. Together with chemokines/cytokines, antigen-specific T helper (Th) cells are activated *via* expansion of Th1 cells. They start to proliferate and exert tissue destructive activities. They further activate cytotoxic T lymphocytes, which stimulate the humoral immune response *via* expansion of Th2 cells and B-lymphocytes (42, 43). Activation of mononuclear phagocytes, another class of APC that also produce pro-inflammatory mediators accompanies the Th1 response. Auto-aggression occurs when immune tolerance, such as T suppressor cells that usually down regulate the overactive immune responses, is lost (41).

The regulatory T cell population (Treg) that inhibits the activation of CD4^+^ and CD25 T effector cells and regulates auto-aggressive T and B cell impacts the potential development of human autoimmune diseases (44). Naive CD4^+^ T cells, upon encountering their cognate, differentiate into effector cells, such as Th1, Th2, Th17, or Treg. These are further characterized by their cytokine production profiles and immune regulatory functions (44):

- Th1 cells regulate antigen presentation and immunity against intracellular pathogens and produce tumor necrosis factor alpha (TNF) and interferon gamma (IFN-γ).
- Th2 cells mediate humoral responses and immunity against parasites. They are important mediators of allergic diseases and produce interleukins (IL)-4, IL-5, and IL-13.
- Th17 cells, which develop *via* an independent lineage from Th1 or Th2 cells, participate in inflammation and autoimmunity processes and express IL-17, IL-17F, IL-21, IL-22, and IL-26.
- Treg mediate immune suppression by secretion of transforming growth factor-β (TGF-β) and IL-10 and express Forkhead box P3 (FOXP3) transcription factor (45).

The innate immune system steers the differentiation of Th cells by providing T cell receptor (TCR), and co-stimulatory signals as well as an appropriate cytokine microenvironment, which ultimately leads to the preferential induction of one specific cell lineage over the other. Of upmost importance for Th1, Th2, and Th17 cell differentiation are IFN-γ, IL-12 and IL-4, while IL-6 and TGF-β potently initiate Th17 differentiation (45).

Further possible mechanisms of autoimmunity include the hygiene hypotheses, which could play an important role in the worldwide increase of autoimmune diseases. It states that pathogens, parasites and commensal microorganisms protect against a variety of autoimmune conditions. Hence, the reduction in infection rates is likely to be one of several factors that has led to an increase in the frequency of certain autoimmune diseases (46). This is paired with changes in diet, especially the increase in food consumption of saponins, lectins, gliadin, and capsaicin, which can increase intestinal permeability, thus leading to increased uptake of endotoxin and inflammation. The increase in autoimmunity may enhance intake of epitopes cross-reactive with self. However, as the increase in autoimmunity did not occur directly after the switch from hunter-gatherer diet to the early agricultural diet, an underlying immunoregulatory deficit was necessary. The hygiene hypotheses does therefore not fully explain the rise in autoimmunity. It seems that genetic, molecular mimicry, and viral hypotheses are incoherent without a major simultaneous environmental change to weaken background immunoregulation, so that certain genotypes, in the presence of certain triggers, can develop these autoimmune diseases (47, 48).

Further mechanisms of co-occurrence other than the genetic-link could be shared, i.e., molecular amino acid signatures in the HLA-DR peptide-binding pocket predisposing to both T1D and AITD. A positively charged (Lys-71, Arg-74) HLA-DR pocket 4 was found to be critical for the development of both T1D and AITD, by accommodating autoantigenic peptides that may initiate both diseases or may facilitate the anchoring of the T-cell receptor to the peptide- MHC II complex (49). While differences exist in the pathogenesis of both diseases, epidemiological data revealed clustering of T1D and AITD in the same individual as well as in families, suggesting a common genetic basis for both diseases. Studies performing family-based association analyses in multiplex families demonstrated a joint genetic susceptibility that involves complex gene-gene and genetic-epigenetic interactions. As the molecular basis for the interactions between susceptibility genes in complex diseases remains unknown, the cumulative effect of increased statistical risk, as well as molecular interactions between susceptibility genes or their products, determine disease phenotype and severity (50–52). Even in countries with low disease incidence familial clustering has been observed (1).

## The Genetic Link

Both T1D and AITD are multifactorial autoimmune endocrine diseases, with several susceptibility genes and environmental factors contributing to disease aetiology (15). Confirmed by genetic studies, a few genes confer risk for developing both AITD and T1D. These genes are denominated as joint susceptibility genes for AP III variant, suggesting that the gene product is implicated in the pathogenesis of both diseases (53, 54). Both whole-genome linkage screening and candidate gene studies have identified these genes (51, 55–57). Comprehensive analysis of gene-gene interactions in T1D showed that the more susceptibility risk alleles an individual carries, the higher the relative risk of developing disease, with a resulting odds ratio of 61. HLA remains the most important contributor to the overall risk, while additional gene interactions are likely to confer either protection or susceptibility (1, 58).

### HLA Genes

**Figure 1:**
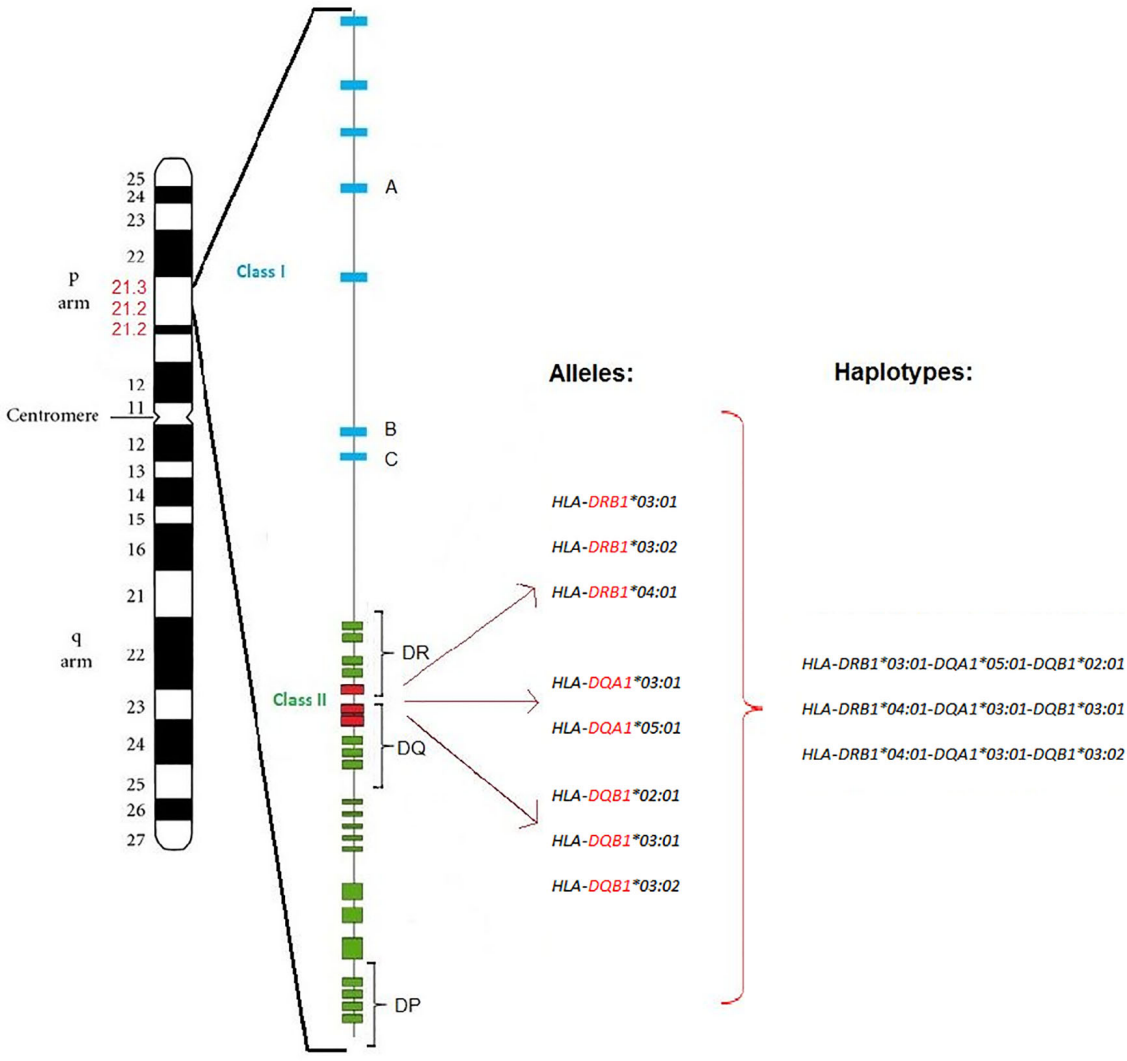
T1D and AITD associated HLA alleles and haplotypes. Position of the HLA gene complex on the p arm of chromosome 6p21 (6p21.1-6p21.3) with around 3,500 kilo bases. The HLA class I region (blue) is located at the telomere side, while the HLA class II region (green) is located at the centromere side. *DRB1*, *DQA1*, and *DQB1* alleles and haplotypes of the HLA class II (red) are associated with both T1D and AITD (AP III variant) in Caucasian subjects. Major susceptibility alleles in Caucasians are *HLA-DRB1-03:01*, *HLA-DRB1-03:02*, *HLA-DRB1-04:01*, *HLA-DQA1-03:01*, *HLA-DQA1-05:01*, *HLA-DQB1-02:01*, *HLA-DQB1-03:01*, and *HLA-DQB1-03:02*. The resulting haplotypes are *HLA-DRB1-03:01-DQA1-05:01-DQB1-02:01*, *HLA-DRB1-04:01-DQA1-03:01-DQB1-03:01*, and *HLA-DRB1-04:01-DQA1-03:01-DQB1-03:02*.

Several genetic studies of patients with autoimmune diseases have shown specific contribution of HLA alleles, mostly HLA class II, to the genetic predisposition to autoimmune diseases. This is crucial for understanding their pathogenesis. There is a correlation between carriage of certain HLA class II alleles and an increased probability of developing the most common autoimmune diseases, including T1D and AITD. Several HLA class I and II alleles have been identified to influence both T1D and AITD, as well as AP (59–62). The HLA gene complex is located on chromosome 6p21 (**Figure 1**) and encodes the major histocompatibility complex (MHC) proteins in humans. MHC class I proteins are heterodimers that consist of a long α-chain containing a transmembrane domain and a short universal β2-microglobulin chain while MHC class II proteins consist of long α- and β- chains carrying extracellular, transmembrane and short cytoplasmic domains. While HLA corresponding to MHC class I present peptides from inside the cell, HLA corresponding to MHC class II present antigens from outside the cell to T-lymphocytes. Class I genes encode for HLA antigens A, B, and C, class II genes encode for α- and β-chains of the heterodimeric HLA class II antigens DR, DP and DQ. Class III genes encode for complement components (63).

HLA class II molecules are synthesized in the endoplasmic reticulum. The immune response develops after the 13–18 amino-acid antigenic peptide is presented by APC, i.e., dendritic cells, B cells or macrophages, using the HLA class II molecule, and is recognized by the respective T-cell receptor on the CD4^+^ T-cell surface (64). HLA-DM, a nonconventional HLA class II molecule, is not polymorphic and cannot interact with antigenic peptides. However, it catalyzes the binding of antigenic peptide to HLA-DR. The HLA class II–peptide complexes are then delivered to the plasma membrane to present peptides to CD4^+^ T cells (65–67).

If an immune response is initiated, CD4^+^ T cells activate B cells for a subsequent production of specific autoantibodies and contribute to the recruitment of macrophages to the immune response. HLA molecules with point substitutions within the antigen-binding groove vary in their efficiency of binding and presentation of self-peptides followed by the initiation of an autoimmune response (68–70). Since HLA class II can present both exogenous and endogenous peptides to CD4^+^ T cells, many biomedical studies have focused on its role in the initiation of autoimmune responses. In patients with autoimmune diseases, such as T1D and AITD, autoantibodies are synthesized and lymphocytes often infiltrate into the target organ, leading to inflammation and even partial destruction. Because antigen presentation and further T cell activation are considered key components of the immune response, studying the peculiarities of antigen presentation as well as the structure and features of HLA proteins in particular is of utmost importance. Interestingly, the autoimmune diseases accompanied by Ab production are typically associated with HLA class II, while the diseases not accompanied by Ab production are more commonly associated with certain HLA class I alleles (71, 72). Although certain HLA Class II molecules may initiate the CD4^+^ T-cell mediated autoimmune response, HLA Class I molecules induce autoantigen-specific CD8^+^ T cell-mediated cytotoxicity, explaining with a plausible immunological rationale, and the genetic association of both HLA Class I and Class II molecules with glandular autoimmunity. This combination has synergistic and complementary effects on various stages of the autoimmune response (73).

HLA-DR3 is the major HLA class II allele contributing to the joint susceptibility for AITD and T1D. HLA haplotypes DR3-DQB1*02:01 and DR4-DQB1*03:02 contribute to AP III variant. The haplotypes HLA-DRB1*03:01-DQA1*05:01-DQB1*02:01 and HLA-DRB1*04:01-DQA1*03:01-DQB1*03:02 are significantly overrepresented in AP III compared to controls (72). The haplotypes for the AITD HT (DRB1*04:01-DQA1*03:01-DQB1*03:02/03:01) and GD (DRB1*03:01-DQA1*05:01-DQB1*02:01), as well as the two haplotypes identified for T1D (HLA-DQA1*03:01-DQB1*03:02 and HLA-DQA1*05:01-DQB1*02:01) are nearly identical. Hence, these HLA alleles and haplotypes confer susceptibility to both T1D and AITD (74–76). Interestingly, in Arabic populations, the haplotype DRB1*03:01-DQB1*02:01 was associated with T1D in individuals from Bahrain, Lebanon, and Tunisia, while the Japanese high-risk haplotypes DRB1*04:05-DQB1*04:01 and DRB1*09:01- DQB1*03:03 as well as the Caucasian haplotypes DRB1*03:01-DQB1*02:01 and DRB1*04:01-DQB1*03:02 were associated with T1D in individuals from Egypt, Morocco, Kuwait, Saudi Arabia, and Algeria (2).

**Figure 2:**
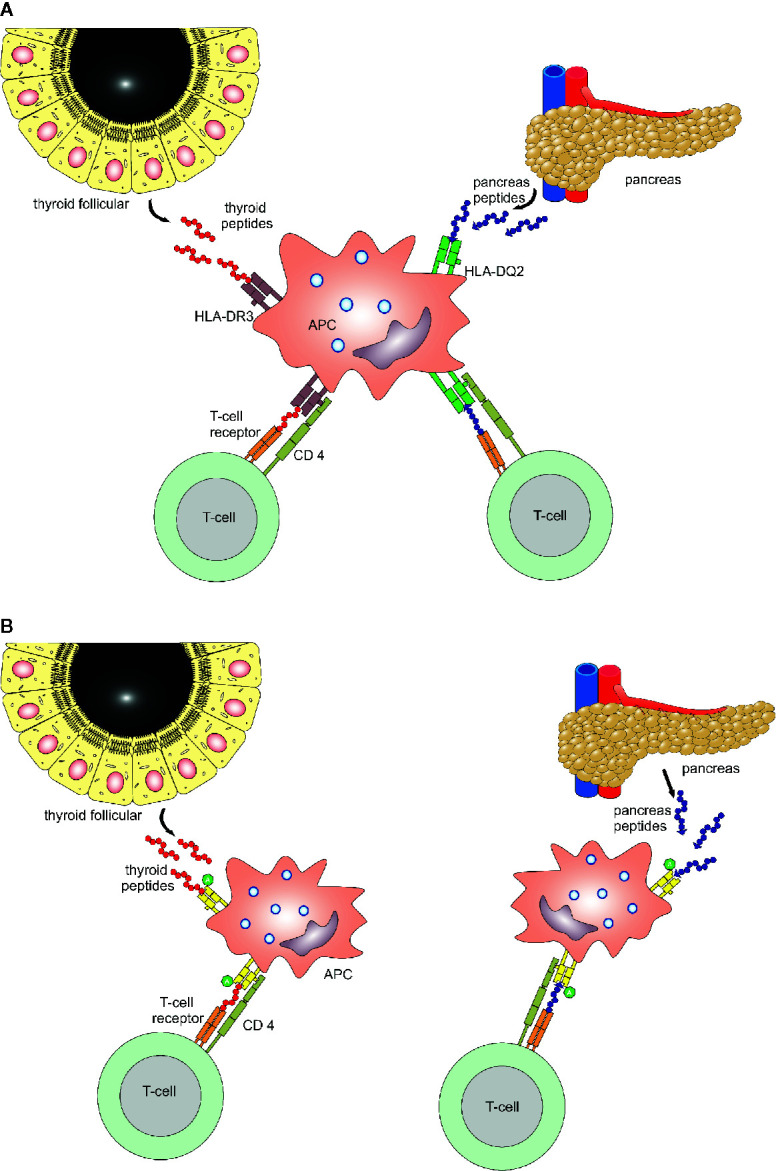
Binding mechanisms of islet and thyroid peptides. Two potential mechanisms for immunologically targeting several glands in patients with polyglandular autoimmunity. Panel **(A)** One APC expresses both pancreas as well as thyroid autoantigens (peptides) which are embedded in pockets with HLA class II molecules and presented to the T cell within the immunological synapse. Panel **(B)** Two APC express either a pancreas or thyroid antigen, however both APC share a common amino acid, which facilitates the anchoring of T cells.

The amino acid at position 57 of the DQ-β chain confers resistance or susceptibility to disease. An aspartic acid on position 57 in both alleles has a protective effect on T1D, while its absence provides susceptibility. In individuals in whom both alleles are non-Asp, the relative risk of T1D has been estimated to be 30 to 107 (1). The specific class II HLA-DR variant containing arginine at position 74 (*DR-1-Arg74*) increases the risk for AITD by blocking thyroglobulin peptides, especially Tg.2098. While bound to *DR-1-Arg74*, it could block continuous T cell activation and the autoimmune response in AITD. Of note, the position 74 in the β chain is exceptionally important, since this amino acid residue is located where the peptide-binding motif of HLA overlaps with the T-cell receptor docking site (77). The *HLA-DR* binding pocket has a unique amino acid signature. Four islet and thyroid peptides (Tg.1571, GAD.492, TPO.758, and TPO.338) were identified with the ability to bind to the *HLA-DR* binding pocket, being presented by antigen-presenting cells and elicited a T cell response. Both thyroid and islet peptides can bind to this flexible binding pocket and induce thyroid and islet specific T cell responses, thus triggering T1D and AITD in the same individual (78). Potential mechanisms how HLA genes activate endocrine autoimmunity and predispose to both T1D and AITD, even if the autoantigenic peptides are distinct, are shown in **Figure 2**. Either one APC expresses both pancreas (islet) as well as thyroid autoantigens (peptides) which are embedded in pockets with HLA class II molecules and presented to the T cell within the immunological synapse, or two APC express either a pancreas or thyroid antigen, however both APC share a common amino acid which facilitates the anchoring of T cells (1, 79, 80).

Another risk allele for both T1D and AITD is *HLA-DQB1* with Alanine at position 57 (Ala57), whereas Aspartic acid (Asp57) is protective. *HLA-DQB1* confers strong susceptibility by Ala57 homozygosity in monoglandular and polyglandular autoimmunity (81). *HLA-DQB1* Ala57 heterozygous women have an increased risk for AITD, whereas males have an increased risk for T1D, revealing sex-dependent increased susceptibility (81). The amino acid residue 57 is responsible for the formation of the DQA1– DQB1 heterodimer. If the aspartic acid is substituted by a neutral amino acid at this position, the HLA molecule will be able to present insulin fragments (82). HLA haplotypes DR3-DQ2 and DR4-DQ8 are associated with the presence of GAD and insulin Ab in patients with recent onset T1D (83). The specific *HLA-DR9/DQ9* subtype might be associated to both T1D and AITD, as it was found in Japanese patients exhibiting both diseases (84). However, the most universal haplotypes positively associated with these diseases are DR3-DQ2 and DR4-DQ8 (85). In the Japanese population, the DRB1-DQB1 haplotypes *DR4, DR8* and *DR9* confer susceptibility to T1D and AITD (86). Moreover, the major susceptible HLA class II haplotype in Japanese associated with AP III was DRB1*04:05-DQB1*04:01 (87), and the haplotype *DRB1∗09:01* was associated with the co-occurrence of T1D and AITD in Japan (88).

Sex alters the *class I HLA-A* association with T1D and AITD (60). In addition, patients with T1D and AITD share a common genetic background of HLA class II antigens DQ2 (DQA1*05:01- DQB1*02:01) and DQ8 (DQA1*03:01-DQB1*03:02) and overlapping functional single-nucleotide polymorphisms of various susceptibility genes involved in the immune regulation (10).

Population based differences are found in the association of HLA with T1D and AITD. Linkage analysis showed that the HLA class II alleles DQ and DR are strongly associated with both diseases. While DR3 is a major risk allele in German, Belgian, Filipino and Chinese Han populations, it does not confer susceptibility in the Japanese population. DR4 is major risk allele in Western Europe, America, South Asia and Africa but is not found in the Chinese Han population. DQA1*03:01, *05:01, and BQB1*02:01 confer risk in Caucasians, while DQB1*03:02 additionally confers risk in USA and DQB1*03:03 in Japan. DQB1*04:01 occurs in lower frequency in Caucasians but is a major risk allele in the Chinese Han population, probably due to its linkage disequilibrium with DRB1*04:05, which with DQB1*03:02 are further major risk alleles in the Chinese Han population (89). In the Northern Indian population, HLA-DRB1*03, DQA1*05, and DQB1*02 were associated with T1D, while worldwide in Caucasians HLA-DR4 (DRB1*04:01/04/05) and DQ8 (DQA1*03:01-DQB1*03:02) are associated with T1D. While in Northern India HLA-DRB1*04 and DRB1*03 were increased in patients with T1D, only the DRB1*03 allele was strongly associated with T1D and DRB1*04 was only increased in combination with DRB1*03. In Southern India however, both DRB1*03 and DRB1*04 contributed equally towards disease predisposition (73).

### Single Nucleotide Polymorphisms in Non-HLA Genes

Additionally to *HLA* genes, several other genes, including the *cytotoxic T-lymphocyte associated protein 4 (CTLA4*, c.+6230G>A, rs3087243) and (*CTLA4*, c.49A>G, rs231775), the *protein tyrosine phosphatase non-receptor type 22 (PTPN22*, c.+1858 C>T, rs2476601), the *interleukin 2 receptor subunit alpha (IL2Ra, c.*A>G rs10795791), the *vitamin D receptor (VDR*, Bsm I rs1544410; Apa I rs7975232; Taq I rs731236), as well as the *tumor necrosis factor (TNF, c.*-863G>A, rs1800630) have been reported as susceptibility genes for T1D and AITD (**Table 1**). Many other genes, such as cluster of differentiation *40* (*CD40), forkhead box P3 (FOXP3), MHC class I polypeptide-related sequence A (MICA), insulin variable number of tandem repeats (INS-VNTR), C-type lectin domain containing 16 (CLEC16A), erb-B2 receptor tyrosine kinase 3 (ERBB3)* gene, the *interferon induced with helicase C domain 1 (IFIH1)* gene and various cytokine genes, are assumed to be involved (90, 91).

**Table 1:** Mutual non-HLA susceptibility genes in Type 1 diabetes and autoimmune thyroid disease.

Gene	Location	Function	Mutations/Polymorphisms	Mutation/Polymorphism Phenotype
**CONFIRMED**				
***CTLA4***	2q33	Encodes a receptor that is expressed on T cells and is a negative regulator of T-cell activation.	+6230G>A (rs3087243) 49A>G (rs231775)	CTLA4 function or expression is affected by the threonine-to alanine substitution in the signal peptide of the CTLA4 protein, which leads to less efficient glycosylation in endoplasmic reticulum and reduced surface expression of CTLA4 protein. Increases susceptibility to T1D, AITD, and AP.
***PTPN22***	1p13	Encodes lymphoid tyrosine phosphatase, a strong inhibitor of T-cell activation, expressed in B and T lymphocytes. Inhibits T lymphocyte antigen receptor signaling pathway	+1858C>T (rs2476601)	Polymorphisms lead to increased T-cell activation and enhanced susceptibility to T1D, AITD, and AP.
***IL2Ra***	10p15	Differentiation factor actively suppresses auto-reactive T cells *via* CD25 and regulates function of natural killer cells, B cells and Treg.	CD25 (rs10795791)	Polymorphisms in the CD25 gene region might affect function of Treg. Increased susceptibility to T1D and AITD, especially GD
***VDR***	12q13.11	Expressed on immune cells and directly inhibits activated T cells. Reduces production of pro-inflammatory cytokines (IFNγ).	Bsm I (rs1544410) Aps I (rs7975232) Taq I (rs731236)	Polymorphisms lead to increased T-cell activation and enhanced susceptibility to T1D.
***TNF***	6p21, within HLA class III region of MHC.	Located on chromosome 6p21, within HLA class III region of MHC.	-308 (rs1800629)	Increased transcription and production of TNF protein through polymorphisms in promoter region, higher levels of TNFα transcription facilitate inflammatory response in autoimmunity. Increases susceptibility to T1D and AITD.
**SUSPECTED**				
***CD40 Gene***	20q13.12	Influences both humoral and cell-mediated immune responses.	−1T>C (rs1883832)	Polymorphisms in the Kozak sequence are associated with autoimmunity, especially AITD.
***FOXP3***	p arm of X chromosome (Xp11.23)	FOXP3 transcription factor occupies the promoters for genes involved in regulatory T-cell function, controls regulatory T-cell differentiation, and is considered the master regulator of Treg development and function.	-2383C>T (rs3761549)	Polymorphisms might affect function of Treg hence increasing susceptibility to T1D and AITD.
***MICA***	p arm of chromosome 6 (6p21.33)	Encodes highly polymorphic cell surface glycoprotein. Protein expressed in two isoforms (MICA1 and MICA2), by alternative splicing. MICA protein is a ligand for receptors on natural killer cells and the stress-induced antigen is broadly recognized by intestinal epithelial T cells.	MICA*A5 variant	Polymorphisms increase susceptibility for organ-specific autoimmune diseases.
***INS-VNTR***	11p15	Translation of pre-proinsulin, precursor of mature insulin. VNTR region of 14 to 15 bp consensus sequence upstream of insulin gene in insulin promoter.	Short class I: (26–63 repeats), Intermediate class II: (64–139 repeats) Larger class III: (140–210 repeats)	Polymorphisms might affect translation of pre-proinsulin, and production mature insulin. Increased susceptibility for T1D in AP type III.
***CLEC16A***	16p13	Encodes a member of the C-type lectin domain containing family.	rs12708716	Polymorphisms increase susceptibility for organ-specific autoimmune diseases, T1D and AITD
***ERBB3***	12q13	Encodes a member of the epidermal growth factor receptor (EGFR) family of receptor tyrosine kinases.	rs2292399	Polymorphisms increase risk for organ-specific autoimmunity, especially T1D and AITD.
***IFIH1***	2q24.3	Belongs to the pattern recognition receptor (PPR) family and is a sensor of double-stranded RNA, initiating antiviral activity through IFN production.	A946T (rs1990760)	Polymorphisms are associated with both T1D and AITD.
***IL1R1***	2q14.2	Encodes a competitive inhibitor of interleukin-1–induced proinflammatory activity	VNTR	Polymorphisms are associated with autoimmunity, especially AITD.
***IL4 Genes***	5q31.1	Is involved in the regulation of immune and inflammatory responses.	-590C>T	Polymorphisms are associated with autoimmunity, especially AITD.

CTLA4, cytotoxic T-lymphocyte associated protein 4; PTPN22, protein tyrosine phosphatase non-receptor type 22; IL2Ra, interleukin 2 receptor subunit alpha; VDR, Vitamin D receptor; TNF, Tumor necrosis factor; CD40, cluster of differentiation 40; FOXP3, forkhead box P3; MICA, MHC Class I Polypeptide-Related Sequence A; INS-VNTR, insulin variable number of tandem repeats; CLEC16A, C-Type Lectin Domain Containing 16; ERBB3, Erb-B2 Receptor Tyrosine Kinase 3 gene; IFIH1, interferon induced with helicase C domain 1; IL1R1, interleukin-1 receptor antagonist; IL4, interleukin 4; GD, Graves’ disease.

A major and confirmed susceptibility gene across different ethnic groups, for both T1D and AITD, is the *CTLA4* gene. Being located on chromosome 2q33, it encodes a receptor expressed on T cells and serves as a negative regulator of T-cell activation. *CTLA4* is involved in the interaction between T lymphocytes and APC. APC activate T lymphocytes by presenting an antigenic peptide bound to a HLA class II protein on the cell surface to the T lymphocyte receptor (92). On the surface of CD4^+^ T lymphocytes, co-stimulatory signals on the APC surface interact with receptors, such as CTLA4 during antigen presentation. *CTLA4* downregulates T lymphocyte activation (93). Also related to autoimmunity is a 3’ untranslated region (3’ UTR), (AT)_n_ microsatellite polymorphism with longer and shorter repeats of AT. Longer repeats are associated with decreased inhibitory function of *CTLA4* and a reduced control of T cell proliferation by correlating with a shorter half-life of the CTLA4 mRNA than shorter repeats (94). Both single-nucleotide polymorphisms (SNP) or rare variants +6230G>A (rs3087243) and 49A>G (rs231775) promote development of autoimmunity, e.g., T1D and AITD by decreasing CTLA4 function. In patients with T1D and AITD (AP III), the *CTLA4* G/G genotype of the +6230 G>A SNP is increased significantly (38, 95). In families with both T1D and AITD, a preferential transmission of the G allele of the *CTLA4* 49A>G SNP in exon 1 can be seen. The 49A>G SNP in the signal peptide of the CTLA4 protein results in a threonine-to-alanine substitution, leading to a less efficient glycosylation in the endoplasmic reticulum and reduced surface expression of the CTLA4 protein. This results in increased T-cell activation by affecting *CTLA4* function and/or expression (37, 38).

Located on chromosome 1p13, the *PTPN22* gene encodes the lymphoid tyrosine phosphatase (LYP), one of the strongest inhibitors of T cell activation. It inhibits the T lymphocyte antigen receptor-signaling pathway by binding to protein kinase Csk and limiting the response to antigens. It is expressed in immature and mature B and T lymphocytes. LYP, associated with the molecular adaptor protein CBL, regulates CBL function in the T cell antigen receptor signaling pathway, as it also binds to Csk, limiting the response to antigens (10, 37, 38). The minor T allele of the +1858 (rs2476601) C>T transition was observed to be associated with T1D, AITD, and AP III (96). The SNP causes a tryptophan for arginine substitution in the LYP protein at codon 620. Distinct isoforms of the protein are encoded through two transcript variants by alternative splicing of this gene (97–99). In addition, patients carrying the minor T allele of the *PTPN22* +1858C>T SNP, had a twofold increased frequency of the HLA-DRB1*03 allele (57). While the minor T allele is known to be involved in altered T lymphocyte activation, a novel SNP in the promoter region of the *PTPN22 gene G1123C*, has been found to be associated with both T1D and AITD in Asian patients (98). Additional polymorphisms may also be causative (54).

The *IL2Ra* gene encoding CD25 is located on chromosome 10p15 and impacts production and function of regulatory T cells, actively suppressing autoreactive T cells in the periphery as a differentiation factor and *via* CD25. It regulates the function of natural killer cells and B cells and plays an important role in the development and function of Treg. A polymorphism in the CD25 gene region (rs10795791) thereby influences the development of the autoimmune diseases and is known to be associated with both T1D and AITD, especially GD (55, 100).

The *VDR* gene is expressed on immune cells and reduces the production of pro-inflammatory cytokines by directly inhibiting activated T cells. Vitamin D3 exerts its immune modulatory function through suppression of activated T cells, resulting in improvement of phagocytosis and suppression of gamma-interferon (IFNγ) production. It therefore may reduce the occurrence of T1D in humans. Three SNPs, BsmI (rs1544410), ApaI (rs7975232), and TaqI (rs731236), have been associated with T1D (101–103), while the BsmI and TaqI polymorphisms are also significantly associated with an increased AITD risk (104). Also, the VDR polymorphism Fokl has been associated with both T1D and AITD in the Brazilian population (105, 106).

The *TNF* gene is located within the HLA class III region of the MHC between HLA-B loci of class I and HLA-D loci of class II on chromosome 6p21. It encodes the pro-inflammatory cytokine TNF. Both the uncommon A allele of the G>A genotype of the -308 (rs1800629) SNP and the C/C genotype in the promoter region of the gene are associated with increased transcription and production of the TNF protein, which has been both implicated in the pathogenesis of autoimmune diseases as well as confers susceptibility to both T1D and AITD. The endogenous production of TNF is influenced by TNF promoter polymorphisms, thereby affecting messenger RNA (mRNA) and protein expression levels. Higher levels of TNF transcription may facilitate the inflammatory response in autoimmunity (91, 107). These SNP show a strong association with the occurrence of T1D and AITD in Asian and Caucasian populations (54, 108). Finally, HLA-DRB1*03 and *TNF* -308*A alleles were strongly associated in patients with AP III. These findings indicate similar immunogenetics of T1D and AITD (109–111).

Further genes are still a matter of discussion regarding their contribution to T1D and AITD. The *CD40* gene-encoded protein also belongs to the TNF receptor superfamily and is mainly expressed in B-lymphocytes, monocytes, thyrocytes, orbital connective tissue, macrophages and dendritic cells, while the CD40 cell surface receptor is expressed on the surface of mature B cells, but not on plasma cells. CD40 influences both humoral and cell-mediated immune responses by interacting with the CD40 ligand on T cells (112). The CD40 ligand (CD40L) binds to the CD40 receptor and is predominantly expressed by activated CD4^+^ T cells, thus activating B-cells and other APC (113). The interaction of CD40-CD40L is vital for the activation of humoral immunity through triggering B cells and production of Ab. CD40 was associated with uncontrolled HLA class II expression and intercellular adhesion molecule 1 (ICAM-1) overexpression in the thyroid follicular cells of patients with AITD (114). Thyroid follicular cells might be able to function as APC under special circumstances (115). The CD40 −1 T>C SNP (rs1883832) in the Kozak sequence is associated with AITD, especially GD (116–118).

CD247 codes for CD3-zeta, a component of the TCR-CD3 signaling complex on T cells. CD3-zeta functions as an amplifier of TCR signaling and the CD3-zeta tyrosine phosphorylation is one of the first events occurring after TCR engagement (119).

The *FOXP3* gene, located on the p arm of the X chromosome (Xp11.23), modulates the differentiation of regulatory T cells. It contains 11 coding exons and belongs to the forkhead/winged-helix family of transcriptional regulators. The FOXP3 transcription factor controls Treg differentiation and is considered the master regulator of Treg development by occupying the promoters for genes involved in Treg function. Genetic variants through mutations of the FOXP3 regulatory pathway reduces function and affects thymocytes developing within the thymus that during thymopoiesis are transformed into mature Tregs, promoting the development of autoimmunity (54, 90, 120). Both a haplotype consisting of 25 repeats of a microsatellite on allele 10 and the T allele of a C>T SNP at position -283 (rs1883832) were related with AP III. Because the microsatellite is located past the zinc finger domain of the *FOXP3* gene, it affects downstream splicing, thereby impeding the function of the gene (121).

*MICA* is located on the p arm of chromosome 6 (6p21.33) and an associated locus within the MHC region. It encodes a highly polymorphic cell surface glycoprotein expressed in two isoforms formed by alternative splicing, MICA1 and MICA2. MICA2 is lacking exon 3. The MICA protein is expressed in epithelial and intestinal cells and is a ligand for receptors on the surface of natural killer cells. It acts as a possibly stress-induced antigen that is broadly recognized by intestinal epithelial T cells, binding to CD8 T cells carrying the integral membrane protein receptor natural killer group 2, member D (NKG2D) as well as natural killer cells. When engaged, the NKG2D–MICA complex results in the activation of T cell responses and natural killer cells against epithelial stressor cells expressing MICA on their surface. It is therefore also connected to organ-specific autoimmune diseases such as T1D and AITD (54, 90, 122, 123).

The *INS gene* region, encoding Insulin, is located on chromosome 11p5. Based on the number of the VNTRs, three classes are differentiated. An association of the *insulin VNTR class I alleles* with AP III has been found (51). A short class I *VNTR* penta-allelic 86-bp tandem repeat in the regulatory 5ʹ-UTR and a longer class III *VNTR* 600 bp tandem repeat polymorphism are related to lower and higher INS expression in the thymus, respectively (54, 90, 124). As insulin binds to insulin- like growth factor 1 receptors and exert its functions and the insulin-like, growth factor 1 receptor overlaps with TSH-R signaling in AITD, DNA alternations affecting the insulin expression influence the TSH-R signaling in AITD.

The *CLEC16A* gene, located on chromosome 16p13, contains a C-type lectin domain. The encoded protein is detected in immune cells, is implicated in pathogen recognition, and might predispose for immune mediated diseases e.g. T1D and AITD (125). The SNP rs2903692 shows a G>A transition significantly associated with both T1D and AITD, with the G allele increasing the risk for AP III (56).

The *ERBB3* gene is located on chromosome 12q13. The SNP rs2292399 in intron 7 of ERBB3 has been shown to be associated with AITD and T1D with the *A allele* increasing the risk for AP III (56).

The *IFIH1* gene, located at 2q24.3 chromosome, belongs to the pattern recognition receptor (PPR) family and is a cytosolic RNA sensor. The gene product, as a sensor of double-stranded RNA, initiates antiviral activity through the induction of IFN regulatory factors 3 and 7, leading to IFN production and apoptosis of virally infected cells, also promoting innate and adaptive immune responses. The SNP *rs1990760* is a non-synonymous polymorphism, an alanine to threonine amino acid change at codon 946A>T, within the *IFIH1* coding region located in the HNF-3b binding site. The polymorphism is associated with both T1D and AITD, especially GD. The correlation between viral infections and development of autoimmune diseases such as T1D and AITD makes *IFIH1* a good susceptibility gene candidate (126–129).

Cytokine genes are also potential candidate genes for development of autoimmunity, especially AITD, since they are involved in the regulation of immune and inflammatory responses (130). The *interleukin-1 receptor antagonist* (*IL-1RA)* gene, located on chromosome 2q14.2, encodes a competitive inhibitor of interleukin-1–induced pro-inflammatory activity. *IL1R1* modulates several interleukin-1–related immune and inflammatory responses and inhibits the activities of interleukin 1, interleukin 1-α and interleukin 1-β. A *VNTR in intron 2* within the *IL1R1* gene has been associated with AITD (131, 132). Genes encoding *interleukin 4* (*IL4*), located on chromosome 5q31.1, are also involved in the regulation of immune and inflammatory responses. IL4 is produced by activated T cells and acts as a pleiotropic cytokine with immunomodulatory functions and is a ligand for the IL-4 receptor. A polymorphism at position −590C>T in the *IL4* gene is associated with AITD (54, 133).

A list of susceptibility genes from several international publications obtained by a NCBI PubMed (https://pubmed.ncbi.nlm.nih.gov/) search for T1D, AITD, and the co-occurrence of both diseases from various populations worldwide is shown in **Table 2**. In detail, in Japan, CTLA4 is strongly associated with T1D or AITD but not with AP III (87, 136). Furthermore, ERBB3, CLEC16A, and CTLA4 are associated with the co-occurrence of thyroid autoimmunity and T1D in the Japanese population (56, 87, 88). CTLA4, PTPN22, IFIH1, INS, CD247, and NAA25, are associated with both T1D and AITD in Sweden (119). The VDR polymorphism FokI is associated with both T1D and AITD in the Brazilian Hispanic population (105). The chemokine receptor type (CCR)5 - 32 bp deletion (Δ32) is associated with both diseases and with celiac disease in Poland (141). Finally, the signal transducer and activator of transcription (STAT)-4 is associated with both T1D and AITD in Korea (140), SESN3 with AITD in the Chinese Han population (139) and SLC26A4 is associated with AITD in the Tunisian population (138). Non-HLA genes like CTLA4 and PTPN22, which have been associated with both T1D and AITD in many Caucasian populations could only be associated with either T1D or AITD in certain Asian and Arab populations (2, 135, 137). Differences of association could also be found in IFIH1 and INS. Interestingly, population differences of IFIH1 susceptibility were also found within Caucasians populations in Europe (134, 135). This raises the question of how strong the population-based differences actually are and if they only occur between populations of different races or even within populations of the race.

**Table 2:** List of population based susceptibility genes for T1D, AITD, and AP subtype III variant.

Gene	Diseases	Country	Ethnecity	Reference
**CTLA4**	T1D	Middle East & North Africa	Arab	Hatem Zayed, 2016 (2)
	T1D	Poland	Caucasoid	Hanna Borysewicz-Sanczyk, 2020 (134)
	T1D	Great Britain	Caucasoid	Suna Onengut-Gumuscu 2015 (135)
	AITD	Great Britain, China	Caucasoid, Asian	Yul Hwangbo, 2018 (136)
	T1D AITD	Japan	Asian	Takuya Awata, 2008 (56)
	T1D AITD	Japan	Asian	Hisakuni Yamashita, 2011 (88)
	T1D AITD	Japan	Asian	Ichiro Horie, 2012 (87)
	T1D AITD	Sweden	Caucasoid	Dan Holmberg, 2016 (119)
**PTPN22**	T1D	Tunisia	Arab	Hatem Zayed, 2016 (2)
	T1D	Great Britain	Caucasoid	Suna Onengut-Gumuscu 2015 (135)
	AITD	Jordan	Arab	Asem Alkhateeb, 2013 (137)
	AITD	Great Britain	Caucasoid	Yul Hwangbo, 2018 (136)
	T1D AITD	Sweden	Caucasoid	Dan Holmberg, 2016 (119)
**IL2RA**	T1D	Japan	Asian	Hisakuni Yamashita, 2011 (88)
	T1D	Great Britain	Caucasoid	Suna Onengut-Gumuscu 2015 (135)
	T1D	Poland	Caucasoid	Hanna Borysewicz-Sanczyk, 2020 (134)
	AITD	Great Britain	Caucasoid	Yul Hwangbo, 2018 (136)
**IFIH1**	T1D	Japan	Asian	Hisakuni Yamashita, 2011 (88)
	T1D	Poland	Caucasoid	Hanna Borysewicz-Sanczyk, 2020 (134)
	T1D	Great Britain	Caucasoid	Suna Onengut-Gumuscu 2015 (135)
	T1D AITD	Sweden	Caucasoid	Dan Holmberg, 2016 (119)
**INS**	T1D	Japan	Asian	Hisakuni Yamashita, 2011 (88)
	T1D	Great Britain	Caucasoid	Suna Onengut-Gumuscu 2015 (135)
	T1D AITD	Sweden	Caucasoid	Dan Holmberg, 2016 (119)
**CLEC16A**	T1D AITD	Japan	Asian	Takuya Awata, 2008 (56)
	T1D AITD	Japan	Asian	Hisakuni Yamashita, 2011 (88)
**ERBB3**	T1D AITD	Japan	Asian	Takuya Awata, 2008 (56)
	T1D AITD	Japan	Asian	Hisakuni Yamashita, 2011 (88)
**CD28**	T1D	Tunisia	Arab	Hatem Zayed, 2016 (2)
**CD40**	GD	Japan	Asian	Naoya Inoue, 2012 (118)
**CD69**	T1D	Great Britain	Caucasoid	Suna Onengut-Gumuscu 2015 (135)
**CD226**	T1D	Great Britain	Caucasoid	Suna Onengut-Gumuscu 2015 (135)
**CD247**	T1D	Tunisia	Arab	Hatem Zayed, 2016 (2)
	T1D AITD	Sweden	Caucasoid	Dan Holmberg, 2016 (119)
**TNF**	T1D	Great Britain	Caucasoid	Suna Onengut-Gumuscu 2015 (135)
**IL2**	T1D	Great Britain	Caucasoid	Suna Onengut-Gumuscu 2015 (135)
**IL10**	T1D	Great Britain	Caucasoid	Suna Onengut-Gumuscu 2015 (135)
**IL21**	T1D	Great Britain	Caucasoid	Suna Onengut-Gumuscu 2015 (135)
**IL27**	T1D	Great Britain	Caucasoid	Suna Onengut-Gumuscu 2015 (135)
**IL7R**	T1D	Japan	Asian	Hisakuni Yamashita, 2011 (88)
	T1D	Great Britain	Caucasoid	Suna Onengut-Gumuscu 2015 (135)
**IL15**	T1D	Tunisia	Arab	Hatem Zayed, 2016 (2)
**VDR**	T1D AITD	Brazil	Hispanic	Denise Barreto Mory et al., 2016 (105)
**NAA25**	T1D AITD	Sweden	Caucasoid	Dan Holmberg, 2016 (119)
**TRB**	T1D	Tunisia	Arab	Hatem Zayed, 2016 (2)
**SLC26A4**	AITD	Tunisia	Arab	Rihab Kallel-Bouattour, 2017 (138)
**SESN3**	AITD	Chinese Han	Asian	Wei Liu, 2018 (139)
**FCRL3**	GD	Japan	Asian	Naoya Inoue, 2012 (118)
	GD	China	Asian	Yul Hwangbo, 2018 (136)
**ZFAT**	HT	Japan	Asian	Naoya Inoue, 2012 (118)
**STAT4**	T1D AITD	Korea	Asian	Yongsoo Park, 2011 (140)
**BANK1**	T1D	Tunisia	Arab	Hatem Zayed, 2016 (2)
**ZAP70**	T1D	Tunisia	Arab	Hatem Zayed, 2016 (2)
**SMOC2**	AITD	Jordan	Arab	Asem Alkhateeb, 2013 (137)
**BACH2**	T1D	Great Britain	Caucasoid	Suna Onengut-Gumuscu 2015 (135)
	AITD	Great Britain	Caucasoid	Yul Hwangbo, 2018 (136)
**IKZF1**	T1D	Great Britain	Caucasoid	Suna Onengut-Gumuscu 2015 (135)
**IKZF3**	T1D	Great Britain	Caucasoid	Suna Onengut-Gumuscu 2015 (135)
**IKZF4**	T1D	Great Britain	Caucasoid	Suna Onengut-Gumuscu 2015 (135)
**AFF3**	T1D	Great Britain	Caucasoid	Suna Onengut-Gumuscu 2015 (135)
**CCR5**	T1D	Great Britain	Caucasoid	Suna Onengut-Gumuscu 2015 (135)
	T1D AITD CD	Poland	Caucasoid	Bartosz Słomiński, 2017 (141)
**CCR7**	T1D	Great Britain	Caucasoid	Suna Onengut-Gumuscu 2015 (135)
**GLIS3**	T1D	Great Britain	Caucasoid	Suna Onengut-Gumuscu 2015 (135)
**SH2B3**	T1D	Great Britain	Caucasoid	Suna Onengut-Gumuscu 2015 (135)
	HT	United States	Caucasoid	Yul Hwangbo, 2018 (136)
**GRP183**	T1D	Great Britain	Caucasoid	Suna Onengut-Gumuscu 2015 (135)
**RASGRP1**	T1D	Great Britain	Caucasoid	Suna Onengut-Gumuscu 2015 (135)
**CTSH**	T1D	Great Britain	Caucasoid	Suna Onengut-Gumuscu 2015 (135)
**DEXI**	T1D	Great Britain	Caucasoid	Suna Onengut-Gumuscu 2015 (135)
**BCAR1**	T1D	Great Britain	Caucasoid	Suna Onengut-Gumuscu 2015 (135)
**FUT2**	T1D	Great Britain	Caucasoid	Suna Onengut-Gumuscu 2015 (135)
**UBASH3A**	T1D	Great Britain	Caucasoid	Suna Onengut-Gumuscu 2015 (135)
**ICOSLG**	T1D	Great Britain	Caucasoid	Suna Onengut-Gumuscu 2015 (135)
**RAC2**	T1D	Great Britain	Caucasoid	Suna Onengut-Gumuscu 2015 (135)
	GD	China	Asian	Yul Hwangbo, 2018 (136)
**TSHR**	GD	Great Britain, China	Caucasoid, Asian	Yul Hwangbo, 2018 (136)
**RNASET2**	AITD	Great Britain, China	Caucasoid, Asian	Yul Hwangbo, 2018 (136)
**CHRNA9**	GD	China	Asian	Yul Hwangbo, 2018 (136)
**MMEL1**	GD	Great Britain	Caucasoid	Yul Hwangbo, 2018 (136)
**PRICKLE1**	GD	Great Britain	Caucasoid	Yul Hwangbo, 2018 (136)
**ITGAM**	GD	Great Britain	Caucasoid	Yul Hwangbo, 2018 (136)
**GPR174**	GD	China	Asian	Yul Hwangbo, 2018 (136)
**SLAMF6**	GD	China	Asian	Yul Hwangbo, 2018 (136)
**ABO**	GD	China	Asian	Yul Hwangbo, 2018 (136)
**LINC01550**	GD	China	Asian	Yul Hwangbo, 2018 (136)
**TG**	GD	China	Asian	Yul Hwangbo, 2018 (136)
**FOXE1**	HT	United States	Caucasoid	Yul Hwangbo, 2018 (136)
**VAV3**	HT	United States, Japan	Caucasoid, Asian	Yul Hwangbo, 2018 (136)
**CAPZB**	HT	United States	Caucasoid	Yul Hwangbo, 2018 (136)
**PDE8B**	HT	United States	Caucasoid	Yul Hwangbo, 2018 (136)
**TRIB2**	AITD	Great Britain	Caucasoid	Yul Hwangbo, 2018 (136)
**LPP**	AITD	Great Britain	Caucasoid	Yul Hwangbo, 2018 (136)
**FAM76B**	AITD	Great Britain	Caucasoid	Yul Hwangbo, 2018 (136)

T1D, Type 1 diabetes; AITD, Autoimmune thyroid disease; GD, Graves’ disease; HT, Hashimoto’s thyroiditis; CD, Celiac disease; CTLA4, cytotoxic T-lymphocyte associated protein 4; PTPN22, protein tyrosine phosphatase non-receptor type 22; IL2RA, interleukin 2 receptor subunit alpha; IFIH1, interferon-induced with helicase C domain 1; INS, insulin gene; CLEC16A, C-Type Lectin Domain Containing 16; ERBB3, Erb-B2 Receptor Tyrosine Kinase 3; CD28, cluster of differentiation 28; CD40, cluster of differentiation 40; CD3z, cluster of differentiation 3z; CD247, cluster of differentiation 247; IL7R, interleukin-7 receptor; IL15, interleukin-15; VDR, Vitamin D receptor; NAA25, N-Alpha-Acetyltransferase 25; TCRβ, T-cell receptor beta; SLC26A4, solute carrier family 26 member 4; CCR)5-(Δ32), Chemokine receptor type (CCR)5 - 32-bp deletion (Δ32); SESN3, Sestrin3; FCRL3, Fc receptor-like protein 3; ZFAT, Zinc Finger And AT-Hook Domain Containing; STAT4, Signal transducer and activator of transcription 4; BANK1, B Cell Scaffold Protein With Ankyrin Repeats 1; ZAP70, Zeta-chain-associated protein kinase 70; SMOC2, SPARC-related modular calcium-binding protein 2; BACH2, BTB domain and CNC homolog 2; IKZF1, IKAROS family zinc finger 1; IKZF3, IKAROS family zinc finger 3; IKZF4, IKAROS family zinc finger 4; AFF3, AF4/FMR2 family member 3; CCR5, C-C motif chemokine receptor 5; CCR7, C-C motif chemokine receptor 7; GLIS3, GLIS family zinc finger 3; SH2B3, SH2B adaptor protein 3; GRP183, G protein-coupled receptor 183; RASGRP1, RAS guanyl releasing protein 1; CTSH, cathepsin H; DEXI, Dexi homolog; BCAR1, BCAR1 scaffold protein, Cas family member; FUT2, fucosyltransferase 2; UBASH3A, ubiquitin associated and SH3 domain containing A; ICOSLG, inducible T cell costimulator ligand; RAC2, Rac family small GTPase 2; TSHR, thyroid stimulating hormone receptor; RNASET2, ribonuclease T2; CHRNA9, cholinergic receptor nicotinic alpha 9 subunit; MMEL1, membrane metalloendopeptidase like 1; PRICKLE1, prickle planar cell polarity protein 1; ITGAM, integrin subunit alpha M; GPR174, G protein-coupled receptor 174; SLAMF6, SLAM family member 6; ABO: ABO, alpha 1-3-N-acetylgalactosaminyltransferase and alpha 1-3-galactosyltransferase; LINC01550, long intergenic non-protein coding RNA 1550; TG, thyroglobulin; FOXE1, forkhead box E1; VAV3, vav guanine nucleotide exchange factor 3; CAPZB, capping actin protein of muscle Z-line subunit beta; PDE8B, phosphodiesterase 8B; TRIB2, tribbles pseudokinase 2; LPP, LIM domain containing preferred translocation partner in lipoma; FAM76B, family with sequence similarity 76 member B.

Further, CTLA4, PTPN22, IL2RA, BACH2, CCR5, SH2B3, and RAC2 are found to be associated with T1D and AITD by various independent genome wide association studies and overlap in our list, indicating a strong common genetic link for T1D and AITD.

## Conclusion

The coexistence of different organ-specific and non- organ-specific autoimmune diseases in the same individual or family could be explained by sharing a common genetic background as well as a defective immune regulation. HLA regions, including DR3, DR4, in association with DQ2 and DQ8 are strongly associated with T1D, AITD, and AP III in Caucasians. HLA haplotypes in patients with AITD (HT and GD), as well as T1D were found to be nearly identical. However, in Arab and Asian populations, HLA susceptibility alleles and haplotypes differ from the ones found in European and American Caucasian populations. Also, population based differences are detected in the association between glandular autoimmunity with both HLA and non-HLA genes. Certain non-HLA susceptibility genes, such as *CTLA4, PTPN22, IL2Ra, VDR*, and *TNF* are involved in the activation of Treg that can react or cross-react with autoantigens. Therefore, polymorphisms in these genes confer further susceptibility to T1D, AITD, and AP III in Caucasians. The contribution of further genes e.g., *CD40, FOXP3, MICA, INS-VNTR, CLEC16A, ERBB3, IFIH1*, and various cytokine genes has not been definitely and/or fully clarified. In conclusion, the combined influence of genetic, epigenetic and environmental factors may lead to the onset of autoimmune disorders in different organs of the same subject or within families. Therefore, genetic screening is useful in patients with monoglandular autoimmunity e.g., T1D and Addison’s disease, as well as their first-degree relatives. In view of the possible long interval between the first manifestation of AP and the subsequent development of further autoimmune endocrinopathies, regular and long-term observation of patients is warranted. Furthermore, screening for autoimmune endocrine diseases is recommended regularly, especially for the offspring of patients with T1D and AITD, and AP III variant.

## Author Contributions

LF conceptualized and designed the study, acquired and analyzed the data as well as drafted the article. GK Project initiation, conception and design, analysis and interpretation of data, drafting and critical revision of the article, as well as approval of the final version to be published. All authors contributed to the article and approved the submitted version.

## Conflict of Interest

The authors declare that the research was conducted in the absence of any commercial or financial relationships that could be construed as a potential conflict of interest.
